# *Helicobacter pylori* CagA Triggers Expression of the Bactericidal Lectin *REG3γ* via Gastric STAT3 Activation

**DOI:** 10.1371/journal.pone.0030786

**Published:** 2012-02-01

**Authors:** Kai Syin Lee, Anastasia Kalantzis, Cameron B. Jackson, Louise O'Connor, Naoko Murata-Kamiya, Masanori Hatakeyama, Louise M. Judd, Andrew S. Giraud, Trevelyan R. Menheniott

**Affiliations:** 1 Murdoch Children's Research Institute, Royal Children's Hospital, Parkville, Australia; 2 Department of Paediatrics, University of Melbourne, Royal Children's Hospital, Flemington Road, Parkville, Australia; 3 Department of Microbiology, Graduate School of Medicine, University of Tokyo, Tokyo, Japan; University of Aberdeen, United Kingdom

## Abstract

**Background:**

Most of what is known about the *Helicobacter pylori* (*H. pylori*) cytotoxin, CagA, pertains to a much-vaunted role as a determinant of gastric inflammation and cancer. Little attention has been devoted to potential roles of CagA in the majority of *H. pylori* infected individuals not showing oncogenic progression, particularly in relation to host tolerance. *Regenerating islet-derived* (*REG*)*3*γ encodes a secreted C-type lectin that exerts direct bactericidal activity against Gram-positive bacteria in the intestine. Here, we extend this paradigm of lectin-mediated innate immunity, showing that REG3γ expression is triggered by CagA in the *H. pylori*-infected stomach.

**Methodology/Principal Findings:**

In human gastric mucosal tissues, *REG3*γ expression was significantly increased in CagA-positive, compared to CagA-negative *H. pylori* infected individuals. Using transfected CagA-inducible gastric MKN28 cells, we recapitulated *REG3γ* induction *in vitro*, also showing that tyrosine phosphorylated, not unphosphorylated CagA triggers *REG3γ* transcription. In concert with induced REG3γ, pro-inflammatory signalling downstream of the gp130 cytokine co-receptor via the signal transducer and activator of transcription (STAT)3 and transcription of two cognate ligands, interleukin(IL)-11 and IL-6, were significantly increased. Exogenous IL-11, but not IL-6, directly stimulated STAT3 activation and *REG3γ* transcription. STAT3 siRNA knockdown or IL-11 receptor blockade respectively abrogated or subdued CagA-dependent REG3γ mRNA induction, thus demonstrating a requirement for uncompromised signalling via the IL-11/STAT3 pathway. Inhibition of the gp130-related SHP2-(Ras)-ERK pathway did not affect CagA-dependent REG3γ induction, but strengthened STAT3 activation as well as augmenting transcription of mucosal innate immune regulators, *IL-6, IL-8* and *interferon-response factor* (*IRF*)*1*.

**Conclusions/Significance:**

Our results support a model of CagA-directed REG3γ expression in gastric epithelial cells via activation of the IL-11/gp130/STAT3 pathway. This response might allow Gram-negative *H. pylori* to manipulate host immunity to favour its own survival, by reducing the fitness of co-habiting Gram-positive bacteria with which it competes for resources in the gastric mucosal niche.

## Introduction

Infection with the Gram-negative bacterium *Helicobacter pylori* (*H. pylori*) is the leading cause of gastric cancer worldwide. Epidemiological studies reveal that strains of *H. pylori* carrying the major protein virulence factor, cytotoxin-associated antigen A (CagA), are associated with an increased risk of gastric cancer compared to strains of *H. pylori* lacking CagA [1]. Current literature indicates that CagA molecules are directly translocated into gastric epithelial cells via a bacterial type-IV secretion system (T4SS), analogous to a ‘molecular syringe’ [2]. Translocated CagA tethers to the inner surface of the plasma membrane [3] and is tyrosine phosphorylated at specific C-terminal Glu-Pro-Ile-Tyr-Ala (EPIYA) repeat motifs [4], [5].

CagA has been shown to interact with several intracellular components of signal transduction pathways, predominantly, though not exclusively, in the tyrosine phosphorylated mode [4], [6], [7], [8], [9], [10]. Src-homology protein tyrosine phosphatase (SHP)2 is an intracellular target and pivotal mediator of CagA. SHP2 is specifically bound by tyrosine phosphorylated CagA and provokes Ras-dependent and independent signalling via the SHP2-(Ras)-ERK (MAP-kinase) cascade. CagA-mediated SHP2 signal transduction leads to deregulation of epithelial cell polarity, characteristically manifested by cell elongation and increased motility, the ‘hummingbird phenotype’ [10]. This cellular response has been attributed to the acquisition of transformed or invasive phenotype, drawing parallels in particular with the pro-oncogenic properties of the epithelial to mesenchymal transition (EMT) [11]. Further evidence arguing in favour of CagA as a pro-oncogenic factor comes from mouse transgenic experiments in which CagA overexpression led to uniform hypertrophy and low frequency, late onset focal tumourigenesis of the gastric epithelium, notably without significant induction of gastritis or atrophy [12]. Thus, CagA clearly deregulates gastric epithelial homeostasis in a cell autonomous manner, however the recruitment of secondary somatic mutations, or additional pro-inflammatory factors is likely required for complete penetrance of oncogenic potential. Additionally, CagA has been shown to increase oncogenic transformation of simian virus (SV)40 large T-antigen and human telomerase reverse transcriptase (hTERT) pre-immortalized gastric epithelial cells by Ras-independent activation of ERK1/2 kinase signalling [13]. While these studies are generally supportive of CagA as a bacterial oncoprotein with activity in mammalian cells, its transforming capability is limited and likely allows cancer progression only in the subset of *H. pylori* infected individuals with pre-existing genetic susceptibility.

IL-6 family cytokine signalling via the glycoprotein (gp)130 co-receptor plays pivotal roles in gastric epithelial homeostasis, inflammation and cancer [14], [15], [16], [17], [18], [19]. In the stomach, signal transduction via gp130 is mediated through two major arms, the aforementioned SHP2-(Ras)-ERK pathway and the Janus kinase (JAK)/signal transducer and activator of transcription (STAT)3 pathway [20]. Augmented gp130/JAK/STAT3 activation has been reported in CagA-positive *H. pylori* dependent gastritis [21], thus arguing for STAT3 hyperactivation driven by CagA. It is well established that constitutive STAT3 activation is both pro-inflammatory and oncogenic [20], [22], and together these studies argue in favour of STAT3 as a factor in CagA-related perturbation of gastric epithelial homeostasis and immunity.

Despite accumulating evidence in support of STAT3 as an intracellular mediator of CagA function [20], [21], [23], [24], a unifying molecular mechanism remains elusive. Interleukin (IL)-11 is a major ligand activator of gastric gp130 signalling and is therefore a logical candidate for CagA-dependent STAT3 activation. Accordingly, Murata-Kamiya *et al.* reported increased IL-11 mRNA on expression microarrays following forced CagA expression in gastric MKN28 cells, though STAT3 activation was not assessed [25]. By contrast, a study based in the laryngeal carcinoma-derived HEp-2 cell line [26] observed IL-6/IL-11 ligand-independent, but IL-6 receptor alpha (IL6Rα) dependent STAT3 signalling by CagA, irrespective of EPIYA tyrosine phosphorylation status [23]. On the other hand, studies by us and others indicate that STAT3 is not activated via IL-6Rα and is triggered principally by IL-11 in the distal stomach, the preferred niche of *H. pylori* [14], [15], [16], [21]. Clarification of the mechanistic and appropriate tissue-specific contexts of CagA-dependent STAT3 signalling, with particular emphasis upon downstream effector genes, would undoubtedly illuminate the biological significance of this host response.

The *Regenerating islet-derived* (*REG*)*3* genes encode an evolutionarily conserved family of secreted, C-type lectins with each member comprised of an approximately 16 kDa carbohydrate recognition domain (CRD) and N-terminal secretion signal. The C-type lectin, REG3γ has been shown to have broad bactericidal activity against commensal Gram-positive bacteria in the intestine by virtue of high affinity binding, via the CRD, to exposed peptidoglycan carbohydrate residues [27], [28], [29]. Consistent with this mode of action, REG3γ has no demonstrable activity against Gram-negative bacteria in which the peptidoglycan layer is concealed beneath the outer cell membrane [27]. In the intestinal mucosa REG3γ expression is directly induced by bacterial contact with host surface epithelial cells [27], [30]. Collectively, these studies identify REG3γ as an inducible and directly antibacterial C-type lectin that functions to restrict potentially harmful mucosal invasion by otherwise beneficial intestinal microflora. In this capacity REG3γ helps to maintain symbiotic host-microbe relationships thus preserving correct intestinal function and homeostasis.

Here, using a cohort of human gastric mucosal tissues and CagA-inducible gastric epithelial cell lines, we show that REG3γ is a transcriptional target of the CagA cytotoxin. Induction of *REG3γ* is unrelated to CagA-dependent deregulation of cell polarity (via inappropriate SHP2/ERK activation) and is instead mediated predominantly by signal transduction by IL-11 via the gp130/JAK/STAT3 pathway. Though not directly required for *REG3γ* transcription, we show that reciprocal CagA-dependent SHP2-(Ras)-ERK signalling subdues pro-inflammatory STAT3 activation, as well as inhibiting downstream regulators of gastric mucosal innate immunity. Though others have reported transcriptional regulation of REG3γ by symbiotic and pathogenic bacteria [27], [30], [31], our study is the first to describe the regulation of a C-type-lectin by an isolated bacterial cytotoxin. The broad significance and applicability of our findings in relation to *H. pylori* biology are discussed.

## Results

### Elevated REG3γ expression in CagA-positive *H. pylori* infection

**Figure 1:**
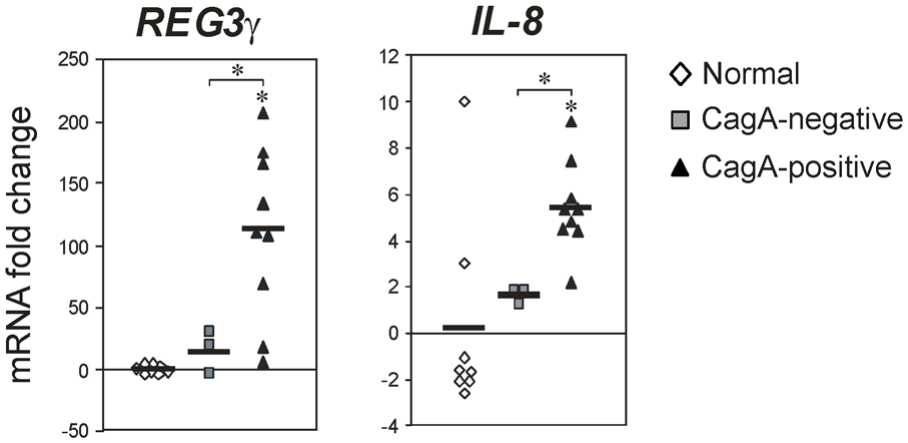
Increased *REG3γ* expression in CagA-positive *H. pylori* infection. Quantitative (Q) RT-PCR analysis of *REG3γ* and *IL8* mRNA levels in human gastric epithelial biopsies encompassing (i) normal (disease-free) controls; gastritis with (ii) CagA-positive *H. pylori*-infection; (iii) CagA-negative *H. pylori*-infection. Scatter plots show the mean mRNA fold-change relative to normal controls (normalized to the internal reference gene *GAPDH*). Black horizontal bars show the mean mRNA fold-change/group. Where present, asterisks show statistical significance (*P*<0.05).

We previously showed in the mouse distal stomach that STAT3 activation is required for the induction of C-type lectins, Reg3β and Reg3γ [15]. In a related study, we also reported STAT3 hyperactivation in CagA-positive, compared to CagA-negative *H. pylori*-infected epithelial tissue from the human distal stomach [21]. Because of the known bactericidal effects of C-type lectins we were intrigued by the potential for regulatory effects of pro-inflammatory STAT3 on REG3γ in the context of CagA-positive *H. pylori* infection. Therefore we determined REG3γ expression levels in gastric epithelial biopsies collected from patients (n = 24) showing histopathologic evidence of gastritis with predetermined *H. pylori* infection and CagA cytotoxin status [21]. Quantitative RT-PCR showed that REG3γ expression was dramatically increased in CagA-positive *H. pylori* infected gastric mucosal biopsies (mRNA fold-change 110.4±23.3; *P*<0.01), but strikingly, was not differentially expressed in CagA-negative *H. pylori*-infected gastric mucosal biopsies (mRNA fold-change 14.8±9.88; *P* = 0.27) compared to disease-free controls. In further support of a CagA dependent effect, expression of the canonical CagA responsive gene, interleukin (IL)-8 [32], [33], was increased in a manner consistent with the changes seen for REG3γ (**Figure 1**). These results indicate that the bactericidal C-type lectin, REG3γ, is overexpressed in CagA-positive, but not CagA-negative *H. pylori* infection in humans.

### Tyrosine phosphorylated CagA triggers *REG3γ* mRNA expression and activates STAT3 signalling

**Figure 2:**
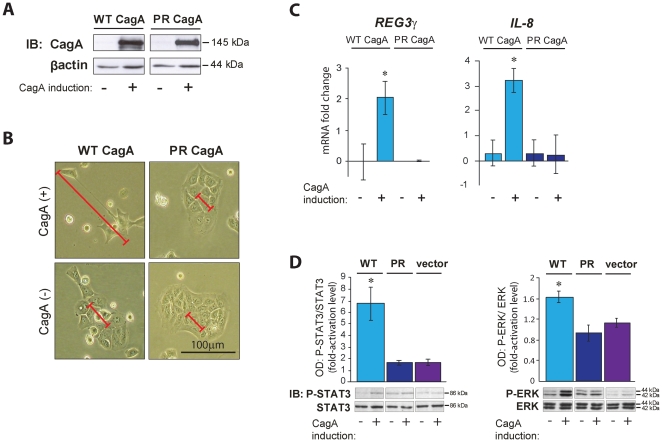
CagA tyrosine phosphorylation triggers REG3γ expression and activates STAT3 signalling. (**A**) CagA immunoblot (IB) analysis of WT-CagA and mutant PR-CagA protein expressing MKN28 cells and matched non-induced control cells. (**B**) Representative photographs of live WT-CagA and PR-CagA expressing MKN28 cells and matched non-induced control cells. Red scale bars indicate the length (along the longest axis) of representative cells in each image. Black scale bar in the lower right hand panel shows 100 µm. (**C**) QRT-PCR analyses of *REG3γ* and *IL-8* mRNA in WT-CagA and PR-CagA expressing MKN28 cells. Histograms show mean mRNA fold change of CagA induced cells compared to non-induced controls. (**D**) Immunoblot (IB) analysis of phosphorylated (P)-STAT3 and P-ERK in WT-CagA, PR-CagA induced, or empty vector MKN28 cells compared to non-induced controls. Histograms show mean fold-change in optical density (OD) of phosphorylated protein bands normalized to total protein bands. Protein bands from one randomly selected replicate experiment are shown (from a total of n = 6 replicates/group). In all cases, protein molecular weights are indicated (kDa). Error bars show ± standard error of the mean (SEM). Where present, asterisks indicate statistical significance (*P*<0.05).

Having identified REG3γ as a likely transcriptional target of CagA in the gastric epithelium we set out to establish an *in vitro* system as an approach to empirically verify this *in vivo* observation, as well as providing a means to functionally dissect transcriptional mechanisms underlying the REG3γ response. With these aims in mind, we employed Tet-OFF MKN28 human gastric epithelial cell lines with stably integrated, doxycycline (DOX)-repressible transgenes, carrying sequences that encode either a wild-type (WT)-CagA protein, a tyrosine phosphorylation resistant mutant (PR)-CagA protein in which serine residues are substituted for tyrosine (Y) in the c-terminal EPIYA motifs [4], [25]. As demonstrated here in WT-CagA transfected MKN28 cells, CagA protein expression is repressed when the cells are exposed to DOX and is induced by the elimination of DOX from the culture medium (**Figure S1**). Prior to commencing our analysis, we first sought to verify that the expressed CagA protein is active in our MKN28 stable expression system. Accordingly we examined the capacity of WT (tyrosine-phosphorylated)-CagA induced cells (**Figure 2A**) to reproduce the previously reported cell polarity defect, known as the ‘hummingbird phenotype’ [4], [34]. Induction of this characteristic loss of cell polarity (indicated by pronounced cell elongation) was clearly acquired in WT-CagA expressing cells but not in matched non-induced control cells. In addition, and consistent with the previously reported dependence of the cell polarity defect upon CagA tyrosine phosphorylation [4], [35], we observed no morphological changes in PR-CagA inducible cells, despite high level accumulation of the mutant PR-CagA protein after removal of DOX (**Figure 2A–B**). These results confirm the integrity of tyrosine phosphorylated CagA protein in our inducible expression system.

To formally substantiate *REG3γ* as a transcriptional target of CagA, we determined *REG3γ* mRNA levels in our CagA-inducible cells. Consistent with our observations *in vivo*, we observed significantly increased *REG3γ* mRNA abundance in WT-CagA overexpressing cells compared to non-induced control cells. By contrast, *REG3γ* mRNA levels were unchanged in PR-CagA overexpressing cells (**Figure 2C**) revealing that the *REG3γ* response is mediated specifically by tyrosine phosphorylated, but not unphosphorylated CagA.

We sought the identity of candidate signal transduction pathways that might mediate CagA-dependent REG3γ induction. In previous work we have shown that CagA triggers the gp130/JAK/STAT3 pathway in human gastric mucosal tissues [21]. We have also shown that gp130/JAK/STAT3 activation is required for induction of *Reg3β*/*Reg3γ* genes in the mouse distal stomach [15]. Consistent with this expectation we observed increased abundance of phosphorylated STAT3 (P-STAT3) in WT-CagA expressing cells however there was no measureable change in P-STAT3 in PR-CagA expressing cells (**Figure 2D**). These results suggested that STAT3 activation is dependent on CagA tyrosine phosphorylation. To consolidate our findings, we next carried out transient transfection of WT-CagA or PR-CagA expression constructs into unmodified (non-stably transfected) AGS and MKN28 gastric epithelial cell lines. In agreement with our data from CagA-inducible cells, we observed that only WT-CagA transfection elicited P-STAT3 accumulation, whereas PR-CagA transfection had either no effect at all, or quantitatively smaller effects than WT-CagA on P-STAT3 levels (**Figure S2**). Collectively, our data argue that intracellular signalling by tyrosine phosphorylated CagA, not unphosphorylated CagA, is the predominant mode of STAT3 activation and REG3γ induction in gastric epithelial cells.

### In gastric epithelial cells IL-11, not IL-6 triggers STAT3 signalling and REG3γ induction

**Figure 3:**
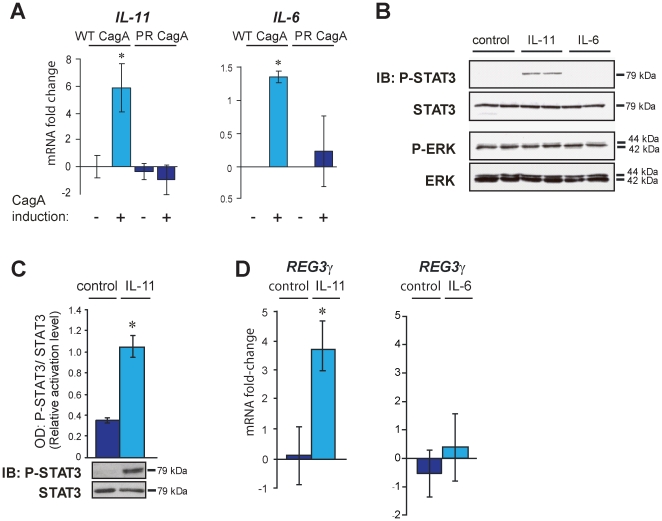
IL-11, not IL-6, induces STAT3 signalling and REG3γ expression in gastric epithelial cells. (**A**) CagA induction assay. Quantitative (Q)RT-PCR analysis of IL-11 and IL-6 in WT-CagA and PR-CagA expressing MKN28 cells. Histograms show mean mRNA fold change of CagA induced cells compared to non-induced controls. Error bars show the standard error of the mean (SEM). Where present, asterisks indicate statistical significance (*P*<0.05). (**B**) Immunoblot (IB) analysis of STAT3 and ERK activation levels (P-STAT3 and P-ERK) in unmodified (non-stably transfected) MKN28 cells exposed to 100 ng/mL recombinant human (rh) IL-11 or rhIL-6. Mock treated (control) cells received 0.22% saline carrier. (**C**) Quantitative immunoblot (IB) analysis of STAT3 activation in MKN28 cells exposed to 100 ng/mL IL-11 and mock-treated control cells. Histograms show mean OD values of P-STAT3 bands normalized to total STAT3 bands. Representative immunoblot images are shown (total of n = 6 replicate cultures/group). Protein molecular weights are indicated (kDa). (**D**) QRT-PCR analysis of *REG3γ* mRNA levels in the MKN28 cells treated with either 100 ng/mL IL-11 (same cell lysates analysed for STAT3 activation in ‘C’) or 10 ng/mL IL-6 (n = 6 replicate cultures/group). Histogram shows the mean mRNA fold-change of rhIL-11/rhIL-6 treated cells compared to mock-treated (saline carrier) controls. Error bars (±SEM). Where present, asterisks indicate statistical significance (*P*<0.05).

The cytokines, IL-11 and IL-6 are major ligand activators of gastric gp130 signalling, via respective interactions with their specific receptor-alpha chains and subsequent heterotrimeric complex formation in combination with gp130 homodimers [20]. Consistent with enhanced STAT3 activation, we found that overexpression of WT-CagA, but not PR-CagA led to increased IL-11 and IL-6 transcription (**Figure 3A**) thus raising the question of whether either, or both of these cytokines might co-ordinate CagA-dependent STAT3 activation and REG3γ expression. To clarify this issue we next investigated whether treatment of normal (non-stably transfected) MKN28 cells with exogenous recombinant human (rh)IL-11 or IL-6 peptide is sufficient for activation of STAT3 and induction of REG3γ in the absence of CagA. Treatment of MKN28 cells with rhIL-11 (100 ng/mL) resulted in rapid P-STAT3 accumulation while, significantly, treatment with rhIL-6 (100 ng/mL) had no effect on P-STAT3 levels. In addition, and consistent with our previously reported *in vivo* analysis of the mouse distal stomach [15], we found no measurable effect of rhIL-11 treatment on ERK activation levels, despite rhIL-11 having a profound stimulatory effect upon parallel STAT3 activation (**Figure 3B**). These results indicate that IL-11, not IL-6, is the cytokine ligand which mediates gastric gp130 signalling by preferential activation of STAT3. Having clarified this issue, we investigated the impact of exogenous rhIL-11 stimulation on REG3γ expression levels in MKN28 cells. We found that rhIL-11 ligand-dependent STAT3 activation (**Figure 3C**) was consistently associated with dramatic upregulation of REG3γ transcription (**Figure 3D**) whilst treatment with IL-6 had no effect on either STAT3 activation (**Figure 3B**) or REG3γ transcription (**Figure 3D**). Our data therefore supports a model in which IL-11, but not IL-6, acts as a key regulator of REG3γ expression in the stomach, very likely via activation of the JAK/STAT3 pathway.

### CagA-dependent REG3γ transcriptional response requires STAT3 and IL-11

**Figure 4:**
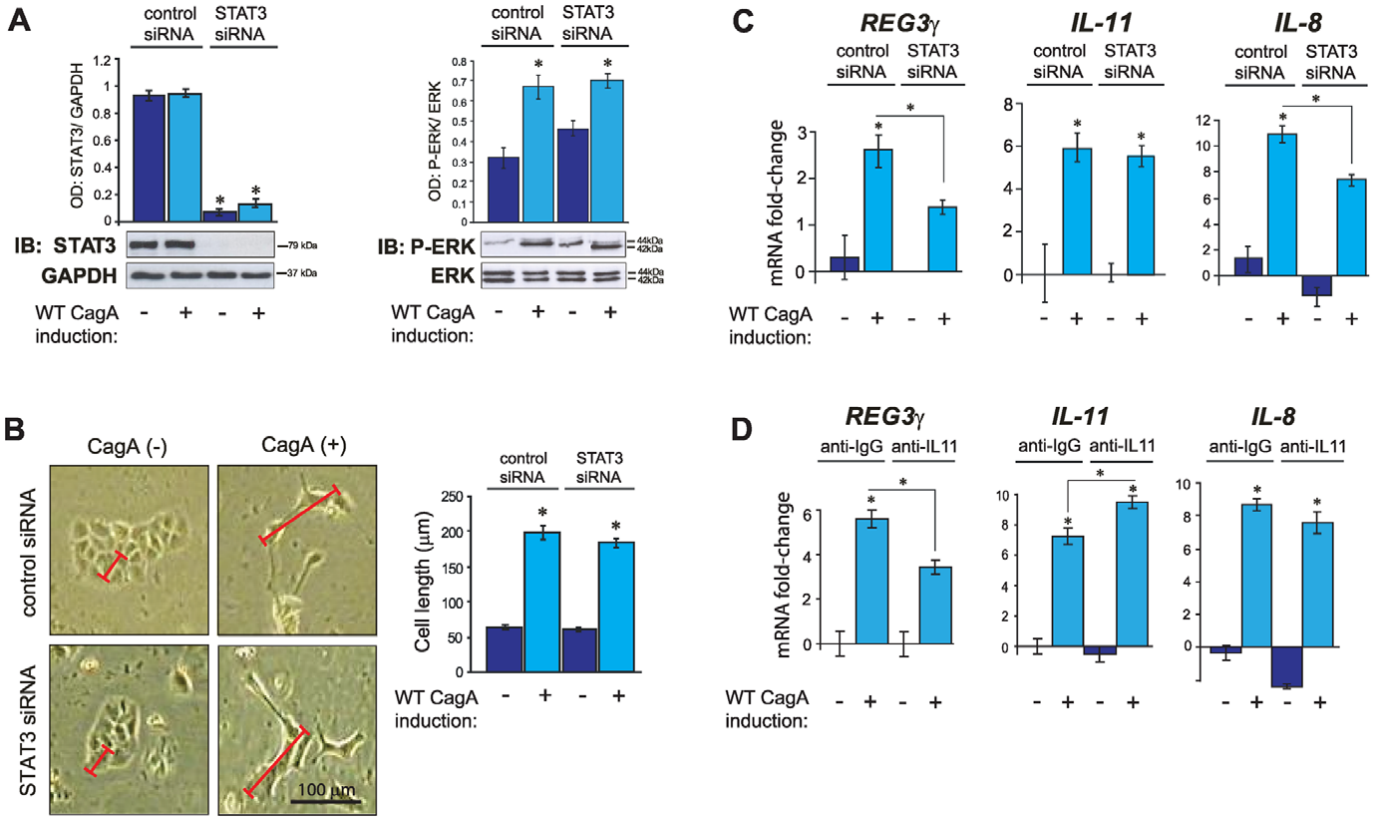
STAT3 RNAi mediated knockdown and IL-11 signalling blockade in CagA expressing MKN28 cells. (**A–C**) RNA-interference (RNAi) mediated STAT3 protein knockdown. (**A**) Immunoblots (IB) of *total* STAT3 and P-ERK in WT-CagA expressing MKN28 cells following transfection with STAT3-specific small interfering (si)RNA or control siRNA. Histograms show mean OD values of *total* STAT3 or P-ERK bands respectively normalized to GAPDH or *total* ERK bands. Representative immunoblot images are shown (total of n = 6 replicate cultures/treatment group). Protein molecular weights are indicated (kDa). (**B**) Morphometric analysis of cell length of the same cultures described in ‘**A**’. Histograms show mean cell length (µm) of WT-CagA expression induced MKN28 cells and non-induced controls following transfection with STAT3-specific siRNA or control siRNA. One randomly selected cell image from each treatment group is shown (total of n = 6 replicate cultures/group). Red scale bars illustrate length (along the longest axis) of representative cells from each treatment group. Black scale bar in the lower right hand panel shows 100 µm. (**C**) QRT-PCR analysis of *REG3γ*, *IL-11* and *IL-8* mRNA expression. Histograms show mean mRNA fold change relative to non-CagA induced, control siRNA transfected cells. (**D**) IL-11 immuno-neutralisation: QRT-PCR analysis of *REG3γ*, *IL-11* and *IL-8* mRNA in WT-CagA expressing MKN28 cells following treatment with anti-human IL-11 specific neutralising antibody or anti-mouse IgG control antibody. Error bars (±SEM). Where present, asterisks indicate statistical significance (P<0.05).

To formally substantiate the role of STAT3 in mediating CagA-dependent REG3γ transcription, a STAT3 specific siRNA was employed in protein knockdown studies. In pilot experiments we confirmed that transfection of a STAT3 specific siRNA led to the near complete ablation of total STAT3 protein, whereas a control siRNA had no effect on STAT3 protein levels (Figure 4A). We found that CagA-related cell polarity defect (hummingbird phenotype) and the associated SHP2/ERK signalling response were unperturbed by STAT3 knockdown (Figure 4A–B). That is, disruption of cell polarity by CagA does not require STAT3. We next assessed the transcriptional effects of CagA in the context of STAT3 protein knockdown. CagA-dependent induction of REG3γ and IL-8 was significantly attenuated in STAT3 deficient, WT-CagA expressing cells (Figure 4C) though, intriguingly, CagA-dependent IL-11 induction was unaffected by the loss of STAT3 (Figure 4C). The latter result suggests that increased IL-11 transcription (and secretion) is a primary function of CagA with STAT3 activation and REG3γ induction occurring secondarily. With this in mind we next employed monoclonal antibodies to selectively block IL-11 ligand/receptor engagement. Consistent with STAT3 protein knockdown studies, IL-11 neutralising antibodies attenuated CagA-dependent induction of REG3γ mRNA (Figure 4D). However we found no significant effect of IL-11 blockade on CagA-dependent IL-8 expression (Figure 4D) or deregulation of cell polarity (data not shown) though clear evidence of positive feedback on IL-11 transcription was detected (Figure 4D). Collectively these data argue that both STAT3 and IL-11 are key components of the (CagA-dependent) gastric REG3γ response, but are dispensable for CagA mediated deregulation of cell polarity.

### CagA-dependent MAP-kinase pathway activation negatively regulates STAT3 and mediators of mucosal innate immunity

**Figure 5:**
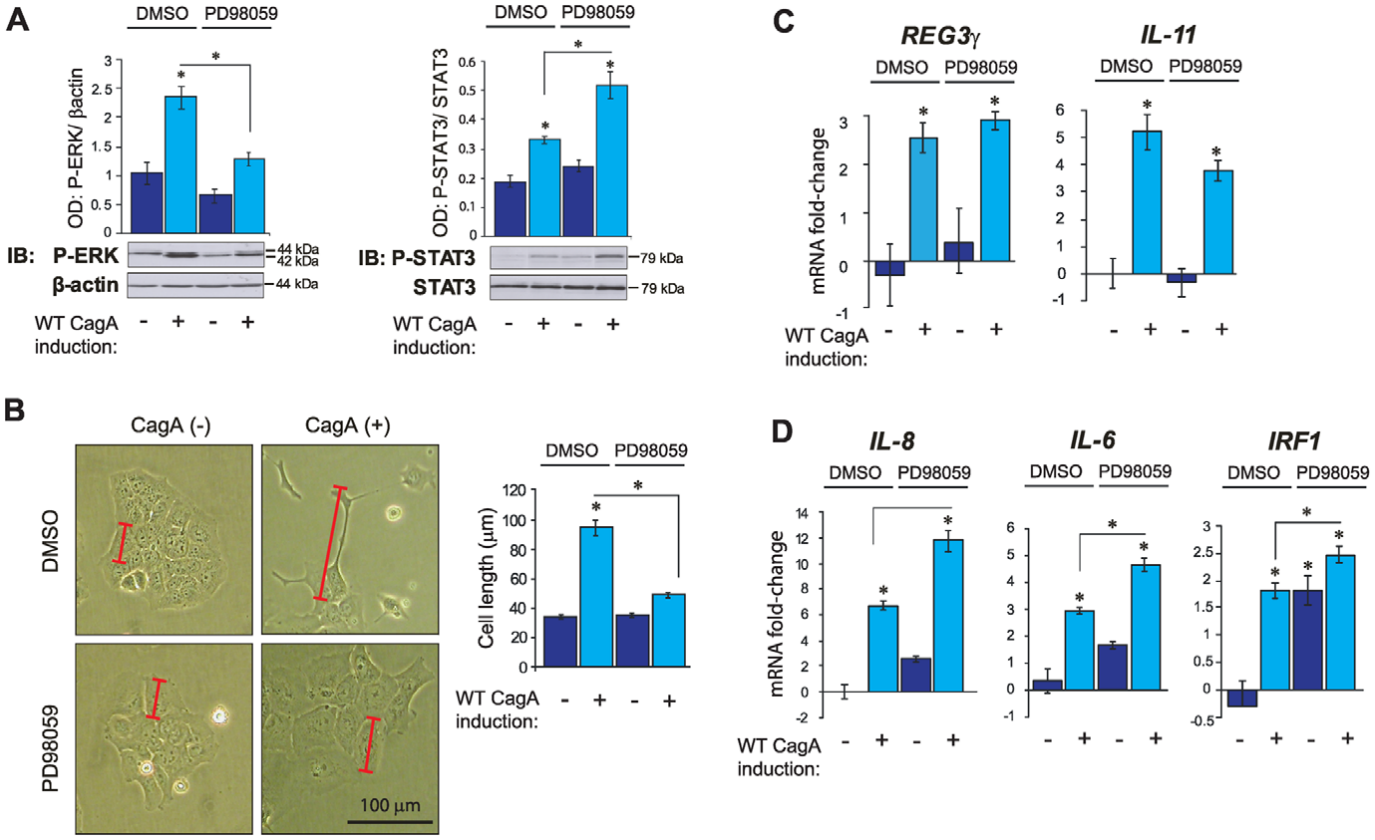
Inhibition of CagA-dependent MAP-kinase pathway activation. (**A**) Immunoblot (IB) analysis of P-ERK and P-STAT3 in WT-CagA inducible MKN28 cells treated with 50 µM PD98059 for 72 hours. Histograms show mean OD values of *total* STAT3 or P-ERK bands respectively normalized to GAPDH or *total* ERK protein bands. Protein bands from one randomly selected replicate experiment are shown (from a total of n = 6 replicates/group used to generate the histogram data). (**B**) Morphometric analysis of cell length. Histograms show mean cell length (µm) of WT-CagA induced MKN28 cells and matched non-induced controls treated with 50 µM PD98059. One randomly selected cell image from each treatment group is shown (n = 6/group). Red scale bars show length (along the longest axis) of representative cells from each treatment group. Black scale bar in the lower right hand panel shows 100 µm. (**C–D**) QRT-PCR analysis of cultures described in ‘**A**’. (**C**) *REG3γ* and *IL-11* mRNA; (**D**) *IL-8*, *IL-6* and *IRF1* mRNA. Histograms show mean mRNA fold changes relative to non-CagA induced, mock treated (DMSO) cells. Error bars (±SEM). Where present, asterisks indicate statistical significance (*P*<0.05).

To comprehensively differentiate between induction of *REG3γ* by either JAK/STAT3 or SHP2/ERK signalling we next assessed the impact of the specific ERK antagonist, PD98059, in WT-CagA expressing MKN28 cells. Treatment with 50 µM PD98059 (empirically determined in dose response experiments as the minimum concentration required for effective ERK inhibition; **Figure S3**) abrogated CagA-dependent ERK signalling (**Figure 5A**), and, as expected on the premise of several published studies [4], [10], completely rescued the cell polarity defect (**Figure 5B**). Strikingly, PD98059 treatment significantly augmented CagA-dependent STAT3 signalling, suggesting the existence of negative regulation by P-ERK (**Figure 5A**). We investigated whether altered SHP2 signalling could account for the apparent release of STAT3 from negative regulation by P-ERK, but found no significant effect of PD98059 treatment on P-SHP2 levels (data not shown). These results indicate that CagA-dependent ERK activation might act to restrain parallel signal transduction via STAT3.

Paradoxically, despite augmented STAT3 signalling, CagA-dependent induction of *REG3γ* mRNA was not significantly increased (though a trend to increase was seen) with PD98059 treatment (**Figure 5C**) suggesting that, in these MKN28 cell lines, maximum transcriptional output had been reached by CagA expression alone. Though difficult to dissect from reciprocal effects on STAT3, these inhibition studies support the view that *REG3γ* induction does not require positive stimulation by CagA-mediated SHP2/ERK signal transduction, and is most likely subject to exclusive regulation by IL-11/gp130/STAT3 signalling. In accordance with the profile of STAT3 activation, transcripts encoding the mucosal immune regulators, *IL-6*, *IL-8* and *IRF1*, were significantly upregulated following blockade of CagA-dependent ERK signalling (**Figure 5D**). These results suggest that CagA induces these genes independently of SHP2/ERK signalling (possibly via, or downstream of STAT3 activation), and/or that ERK-dependent processes may act broadly to subdue (STAT3-related) mucosal immune responses to *H. pylori* infection (**Figure 5D**).

Besides deregulating epithelial cell polarity and triggering innate immunity, CagA has been widely described to alter cell growth kinetics. Whether the cellular growth response to CagA is inhibitory or stimulatory is determined by the polarity status of the recipient epithelial cell [11]. CagA-dependent induction of transcripts encoding key cell cycle regulators including *c-MYC*, C*yclin-D1*, *CyclinE1* and *P21* was partially attenuated following ERK blockade (**Figure S4**). This is intriguing since CagA likely blocks cell cycle progression (**Figure S5**) by generating a dominant anti-proliferative response involving the canonical tumour suppressor genes, *P21* [11], [25] and *Retinoblastoma* (pRB1) (**Figure S6**). These data therefore implicate positive regulation of *P21* (and other cell cycle regulators) by CagA dependent ERK signalling. Taken together our findings also show that REG3γ induction occurs independently of cell cycle activity suggesting that it is separable from the growth effects of CagA. In summary, it seems likely that target genes acting downstream of CagA, which mediate either mucosal innate immunity or growth control are respectively partitioned according to specific regulation by either JAK/STAT3 or SHP-2/ERK signal transduction.

## Discussion

Here we have identified the bactericidal C-type lectin, REG3γ, as a transcriptional target of the principal *H. pylori* cytotoxin, CagA in the human stomach. In addition we show that CagA-dependent regulation of REG3γ requires signalling by the IL-6 family cytokine, IL-11, very likely via the intracellular STAT3 pathway. This REGγ transcriptional response occurs independently of both CagA-dependent deregulation of epithelial cell polarity or signalling via the gp130/SHP2/ERK (MAP-kinase) pathway. On this line, though clearly transducing several immune-related effects of translocated CagA, STAT3 is entirely dispensable for the development of the characteristic CagA-mediated cell polarity defect, the ‘hummingbird phenotype’. This is perhaps unsurprising since CagA-dependent deregulation of cell polarity has been unambiguously attributed to downstream effects on Ras-independent, SHP-2 and MAP-kinase signalling [4], [10]. Nonetheless, we conclude that CagA-mediated gastric REG3γ expression is activated predominantly by IL-11 and STAT3 signalling. Our conclusions are broadly corroborated by other studies showing IL-11 dependent activation of *Reg3β*/*Reg3γ* in the mouse distal stomach as well as overexpression of human REG3 orthologues in concert with elevated IL-11 expression and constitutive STAT3 activation in gastric precancerous lesions [15].

Phosphorylation status is a key determinant of CagA-mediated signalling and transcriptional outcomes, with CagA reported to function predominantly, though not exclusively, in the EPIYA tyrosine phosphorylated mode [4], [6], [7], [8], [9], [10]. Accordingly we found that tyrosine phosphorylated CagA, not unphosphorylated CagA triggered IL-11 expression, STAT3 activation and *REG3γ* mRNA induction. In apparent contradiction to our observations, a recent study reported IL-6Rα dependent activation of STAT3 signalling by CagA, which was also independent of CagA EPIYA motif tyrosine phosphorylation status [23]. The discrepancy between our findings and the work of Bronte-Tinkew *et al.*, in terms of both CagA phosphorylation status and ligand/receptor dependent STAT3 activation mode, is likely due to contrasting differentiated cell types used in the two respective studies. To mimic as closely as possible, the obligate ecological niche of *H. pylori*, we utilized gastric epithelial cell lines, whilst Bronte-Tinkew *et al.* instead used non-gastric, laryngeal carcinoma derived HEp-2 cells. The context of tissue-specificity is a probable extenuating factor in these opposing experimental endpoints. Indeed it is well known that stark mechanistic differences in ligand/receptor mediated STAT3 activation exist between different tissue lineages [36]. From this perspective, it must be acknowledged that our investigation in gastric epithelial cells more accurately recapitulates the tissue-specific STAT3 responses typically encountered by *H. pylori in vivo* than other studies performed in non-gastric cells.

In this study we observed negative regulation of STAT3 by CagA-dependent signalling through the SHP2-ERK pathway. This finding argues that, in addition to effects on epithelial barrier integrity, CagA-dependent MAP-kinase signalling might facilitate *H. pylori* colonisation by restraining pro-inflammatory STAT3 activation and the influence of downstream effector genes. This supposition is well supported by our observation that CagA-dependent upregulation of pro-inflammatory *IL-8*, *IL-6* and *IRF1* transcripts was enhanced in concert with augmented STAT3 signalling after ERK signalling blockade. IL-8 has been well described to promote activation and mucosal infiltration of neutrophils in response to infection with (CagA-positive) *H. pylori* [32], [33], [37]. IL-6 has wide-ranging stimulatory effects on both adaptive and innate immunity and is also a likely participant in the mucosal response to *H. pylori* infection [38], [39]. Similarly, *IRF1* has been described as a *H. pylori* and CagA responsive gene and may be required for full transcriptional activation of *IL-8* [40]. In our study CagA-dependent *IL-8* mRNA induction was partially dependent on STAT3 (*IL-8* expression was significantly attenuated in the presence of STAT3 siRNA). Regulation of *IL-8* transcription by STAT3 has been described in other tissue lineages [41], [42] in addition to our observations here in gastric epithelial cells. Besides mediating CagA-dependent REG3γ induction, STAT3 might also orchestrate broader mucosal immune responses downstream of translocated CagA, the magnitude of which are determined by the strength of counteractive CagA-dependent SHP2-(Ras)-ERK signals.

REG3γ is a specialized C-type lectin, having direct bactericidal activity in the gastrointestinal tract based on peptidoglycan recognition [27], [28], [29]. These studies have established a revelatory paradigm for gastrointestinal biology by elucidating REG3γ as a key homeostatic regulator of intestinal symbiotic host-microbe relationships. Our findings suggest an analogous, but previously undescribed role for REG3γ in the stomach, thereby alluding to the existence of lectin-mediated innate immunity which prospectively modifies the gastric microbiome. Defining the microbial targets of REG3γ in the gastric mucosa will be critical to understanding fully its relationship with *H. pylori* and these must now be elucidated by further studies. This is a pertinent avenue for future research since REG3γ is only directly bactericidal against Gram-positive bacteria [27], [28], [29] and it is unclear how this molecule might interact with, or influence *H. pylori*, a Gram-negative species. We favour a model in which CagA-dependent induction of REG3γ presents a competitive advantage to *H. pylori* at the expense of other gastric microflora (specifically Gram-positive bacteria) which are directly sensitive to the bactericidal properties of REG3γ. Indeed a recent study, showing significantly reduced abundance of Gram-positive *Actinobacteria* and *Firmicutes* species in the *H. pylori*-infected human gastric mucosa [43], provides empirical support for our argument.

The existence of host mechanisms that might indirectly preserve *H. pylori* colonisation necessitates a re-evaluation of the host-pathogen relationship. While undoubtedly oncogenic, the most severe outcome of *H. pylori* pathogenicity, gastric adenocarcinoma, is manifested in a very small minority of infected individuals (∼3/10,000 individuals/year or 2.1% for lifetime infection) [44]. On the other hand, the majority of chronically infected individuals prevail with only superficial gastritis or limited disease progression and do not develop cancer. Intriguingly, a recent study in a mouse infection model suggests that, in some individuals, the early development of tolerance to *H. pylori* may protect against infection-related precancerous disease later in life [45]. Within this framework there is growing debate centred on the notion that *H. pylori* infection is not universally deleterious, and may, in fact be advantageous at least in some instances [46], [47], [48], [49]. Particularly resonant in this regard are recent studies reporting that *H. pylori* infection is protective against gastroesophageal reflux disease (GERD) and paediatric asthma [48], [49], [50], [51]. This emerging evidence has engendered a shift in contemporary thinking towards the concept of *H. pylori* as a symbiotic colonist, thus moving away from the exclusively pathogenic role of conventional understanding. Speculatively, this argues for a cost-benefit scenario whereby the few infected individuals who succumb to intestinal-type gastric adenocarcinoma essentially ‘pay the price’ on behalf of the overwhelming majority that instead benefit from putative ‘immunological balancing’ effects of *H. pylori* infection [48]. As postulated by others [52], the reciprocity of the host-microbe relationship might operate by permitting a mild but tolerable degree of cell lysis arising from low level mucosal inflammation. Conceivably this process would generate nutrient release which, in turn would favour ongoing *H. pylori* colonisation.

In summary, most of what is known about CagA pertains to a much vaunted role as an early determinant of gastric cancer. By contrast, considerably less attention has been paid to possible roles of CagA in the vast majority of chronically infected individuals not showing oncogenic progression [46]. We have provided novel evidence that Gram-negative *H. pylori*, acting via the CagA cytotoxin, directs expression of the Gram-positive specific bactericidal lectin, REG3γ, in gastric epithelial cells and does so by activating the IL-11/gp130/STAT3 pathway. While the functional basis of this response is not entirely clear, we suggest that CagA-directed REG3γ expression may allow *H. pylori* to manipulate host immunity to gain survival advantage, by reducing the fitness of co-habiting Gram-positive bacteria with which it competes for resources in the gastric mucosal niche.

## Materials and Methods

### Ethics statement

Human gastric mucosal biopsies were collected by routine endoscopy under existing human ethics approval from the Royal Melbourne Hospital Human Research Ethics Committee (approval number RMH HREC 2004.176). We have recruited patients to obtain gastric biopsies with pathologist determined *Helicobacter pylori* infection and CagA cytotoxin status as well as non-infected disease-free controls. Written informed consent was obtained for all participants involved in the study.

### Tissue sources

Gastric epithelial biopsies (distal stomach) were obtained from 98 patients of mixed ethnicity undergoing routine gastroscopy at the Western Hospital, Melbourne, Australia, in accordance with Ethics Committee approval. Written informed consent was obtained for all participants involved in the study (*see* **Ethics statement**). *Helicobacter pylori* infection and CagA cytotoxin status was determined as described [21].

### Gene expression

Total RNA was isolated from tissue and cell lines using Trizol (Invitrogen) and contaminant genomic DNA removed with *DNA-free* reagents (Ambion). Primer sequences were designed using primer3 (http://frodo.wi.mit.edu/primer3/) and are listed in the supplemental methods. For quantitative reverse transcription and polymerase chain reaction (QRT-PCR), oligo-dT primed cDNA was synthesised from 1 µg total RNA using Murine Moloney Leukaemia Virus (MMLV) reverse transcriptase (Promega). QRTPCR was performed on an ABI Prism® 7500 Real Time PCR System using SYBR green master mix (Applied Biosystems) according to the manufacturer's protocols. Relative gene expression values were obtained by normalization to the reference gene *GAPDH* using the −2^ΔΔCt^ method, where −2ΔΔCt = ΔCt sample−ΔCt calibrator (Applied Biosystems) as described [53].

### Mammalian cell culture, transfection and RNA interference

Human gastric epithelial Tet-OFF MKN28 cell lines with stably integrated doxycycline (DOX) repressible WT-CagA (WT-A10) and PR-CagA (PR-C2) transgenes, and parental control cells (MKNII) were cultured and manipulated as described [25]. Unmodified (non-stably transfected) AGS and MKN28 human gastric cell lines were maintained in RPMI 1640 medium supplemented with 10% fetal bovine serum (FBS), 50 IU/mL penicillin, 50 µg/mL streptomycin (Invitrogen). For transient overexpression of WT-CagA or PR-CagA proteins, unmodified AGS and MKN28 cells were transfected with 2 µg supercoiled plasmid DNA using FuGENE HD (Roche) according to manufacturer's protocols. After 48 hours cells were washed in ice cold PBS and cell lysates obtained using Trizol (Invitrogen). For STAT3 RNA interference (RNAi) knockdown, CagA-inducible MKN28 cells were grown to ∼70% confluence, then trypsinized and washed three times in prewarmed 2% FBS in PBS, followed by three washes in prewarmed Opti-MEM (Invitrogen). Washed cells (2×10^6^ cells/mL) were electroporated with 4 µg stealth STAT3 siRNA (#46–1468; Invitrogen) or control siRNA. Electroporated cells were plated in 25 cm^2^ flasks and cultured for 72 hours, after which cell lysates were obtained and STAT3 protein knockdown verified by immunoblotting.

### Cytokine stimulation and signal transduction inhibition assays

The source of recombinant human (rh)IL-11 was described previously [21]. Prior to cytokine stimulation, MKN28 cells were seeded in 6-well plates and serum starved by incubation in RPMI 1640 medium supplemented 0.5% FBS for 16 hours. Cell cultures were then incubated with either 100 ng/mL rhIL-11 [21] or 10 ng/mL rhIL-6 (Sigma Aldrich) for 1 hour. To selectively block IL-11 signalling, WT-CagA inducible MKN28 cells were seeded in 25 cm^2^ flasks (5×10^5^ cells/flask) and treated with either 10 ug/mL anti-human IL-11 mouse monoclonal neutralising antibody (#MAB218; R&D Systems) or 10 ug/mL control anti-mouse IgG for 72 hours. For inhibition of ERK1/2 MAP-kinase signalling, cells were treated with the MEK antagonist, PD98059 [54] (50 µM) or mock treated with DMSO carrier for 72 hours. Signal transduction outcomes of cytokine stimulation, antibody neutralisation or pharmacologic inhibition were determined by immunoblotting as described below.

### Immunoblotting

Immunoblotting was performed as we described previously [55]. The sources of primary antibodies are as follows. Rabbit polyclonals: anti-CagA antigen #HPP-5003-9, diluted 1∶1000 (Austral Biologicals); anti-human STAT3 #9132, diluted 1∶1000; anti-human phosphorylated (tyrosine 705) STAT3 #9135, diluted 1∶500; anti-human ERK #9102, diluted 1∶1000; anti-human phosphorylated (threonine 202, tyrosine 204) ERK #9101, diluted 1∶1000; anti-human SHP2 #3752, diluted 1∶1000; anti-human phosphorylated (tyrosine 542) SHP2 #3751S, diluted 1;500; phosphorylated (serine 780) retinoblastoma protein 1 (RB1) #9307, diluted 1∶500; anti-human β-actin #4970, diluted 1∶3000 (all Cell Signaling Technology); anti-GAPDH #ab9485, diluted 1∶3000 (Abcam). Mouse monoclonals: anti-human RB1 #ab24, diluted 1∶1000 (Abcam). Proteins were analysed by quantitative densitometry using Quantity One software (BIORAD), where the optical density (OD) of phosphorylated protein bands was normalized against that of respective total protein bands, GAPDH or β-actin. Unless stated otherwise, results were expressed as mean normalized OD of n = 6 replicate cultures/treatment group.

### Quantitative live cell morphometry

Images of live cultured cells were captured on an inverted microscope using a digital camera. A total of six replicate cultures were analysed for each treatment group. For each replicate/culture flask, three random fields were photographed and for each field, cell length (taken as the distance in µm along the longest cell axis) was determined for three random cells using ImageJ software (http://rsbweb.nih.gov/ij/download.html).

### Statistical analysis

All data expressed as mean ± standard error of the mean (SEM). Comparisons between data groups were made using SigmaStat software (SPSS). T-tests were used for parametric comparisons and where necessary, Mann-Whitney Rank Sum tests were used for nonparametric comparisons. Probability (*P*) values of <0.05 were considered statistically significant.

## Supporting Information

Figure S1 **CagA inducible expression in doxycycline repressible Tet-OFF MKN28 cells.** The Tet-OFF system was used to inducibly express WT-CagA and phosphorylation resistant (PR) CagA mutant cDNAs in human gastric epithelial MKN28 cells. In the Tet-Off system, transcription of the gene of interest is repressed in the presence of the tetracycline analogue, doxycycline (DOX) and maximally expressed in the absence of DOX. In the panel above, stably transfected, WT-CagA inducible Tet-OFF MKN28 cells were treated with a range of DOX concentrations (0–2000 ng/mL) for 24 hours, total protein lysates obtained and immunoblotted (IB) with a specific antibody to CagA. The immunoblot results show that WT-CagA protein expression is induced with decreasing DOX concentration. Similar results were obtained in PR-CagA inducible Tet-OFF MKN28 cells (data not shown). The β-actin immunoblot verifies equivalent total protein loading in all lanes. All experiments described in this study compare cellular responses between non-induced CagA (2000 ng/mL DOX) and fully induced CagA (no DOX treatment).
(TIF)

Figure S2 **STAT3 activation following transient overexpression of CagA protein.** Unmodified AGS and MKN28 cells were transiently transfected with constructs carrying either WT-CagA, or PR-CagA cDNAs driven by the constitutive SR-alpha (SV40/R-U5 T-cell leukaemia virus) promoter fragment, empty vector or mock transfected. Total cellular protein lysates were collected at 48 hours post transfection and immunoblotted for phosphorylated (P)-STAT3, total STAT3 and CagA proteins. Histograms show mean optical densities of P-STAT3 protein bands normalized to total STAT3 protein bands. Protein bands from one randomly selected replicate experiment are shown (from a total of n = 3 replicates/group used to generate the histogram data). Protein molecular weights (kDa) are indicated to the right of the immunoblot images. Error bars (±SEM). Where present, asterisks indicate statistical significance (P<0.05).
(TIF)

Figure S3 **Optimisation of PD98059-mediated ERK inhibition.** Tet-OFF MKN28 WT-CagA inducible cells were treated with different concentrations (0–100 µM) of the MEK (proximal activator of ERK) inhibitor PD98059 for 24 hours. Control cells received an appropriate volume of DMSO carrier. To determine ERK activation levels in response to PD98059 treatment, cell protein lysates were obtained and immunoblotted (IB) for total and phosphorylated (P) forms of ERK. Molecular weight of protein bands is indicated (kDa). Treatment with 50 µM PD98059 was the minimum concentration required for sustained ERK inhibition.
(TIF)

Figure S4 **CagA-dependent induction of cell cycle regulator genes following ERK signalling blockade.** Quantitative (Q) RT-PCR analysis of *P21*, *cMYC*, *CYCLIN-D1* and *CYCLIN-E1* mRNA expression in CagA expressing (+) and non-induced control cells (−) treated with 50 µM PD98059 or mock treated (DMSO). Histograms show the mean mRNA fold-change compared to non-induced, mock treated cells. Error bars (±SEM). Where present, asterisks indicate statistical significance (P<0.05).
(TIF)

Figure S5 **CagA tyrosine phosphorylation leads to growth inhibition of gastric epithelial cells.** CagA induction assay showing CFSE proliferation profiles generated by flow cytometric analysis of WT-CagA and PR-CagA expressing cells together with empty vector control cells. The uppermost panels plot initial numbers of CFSE labelled cells at 0 hours (Parent generation), whilst the middle and lower panels plot cell numbers against CFSE intensity signals for non-induced controls (−) and CagA expression induced (+) cells respectively after 72 hours in culture. Successive cell generations from 1(parent) to 7 are indicated by the colour key. White and black arrowheads in the WT-CagA panels respectively illustrate the differential in CFSE fluorescence intensity between induced cells which have arrested in generation 5 and non-induced cells which have continued to divide.
(TIF)

Figure S6 **CagA tyrosine phosphorylation deregulates the cell cycle modulators RB1 and P21.** (**A**) CagA induction assay showing immunoblot (IB) analysis of Retinoblastoma protein (RB)1 activity. Histograms show mean fold-change in optical density (OD) of phosphorylated (P)-RB1 protein bands normalized to total RB1 protein in WT-CagA and PR-CagA expressing MKN28 cells compared to non-induced control cells. Blot images from one experiment are shown (from a total of n = 6 replicate cultures/group used to generate the histogram data). (**B**) QRT-PCR analysis of *P21* mRNA expression. Histograms show the mean mRNA fold-change in WT-CagA and PR-CagA expressing cells compared to the respective non-induced controls. Error bars (+/−SEM). Where present, asterisks indicate statistical significance (P<0.05).
(TIF)
