# Supplementary figures and images for: Helicobacter pylori CagA Triggers Expression of the Bactericidal Lectin REG3γ via Gastric STAT3 Activation
Source: PLoS One. 2012 Feb 1;7(2):e30786. doi: 10.1371/journal.pone.0030786 (PMC3270022; doi:10.1371/journal.pone.0030786)

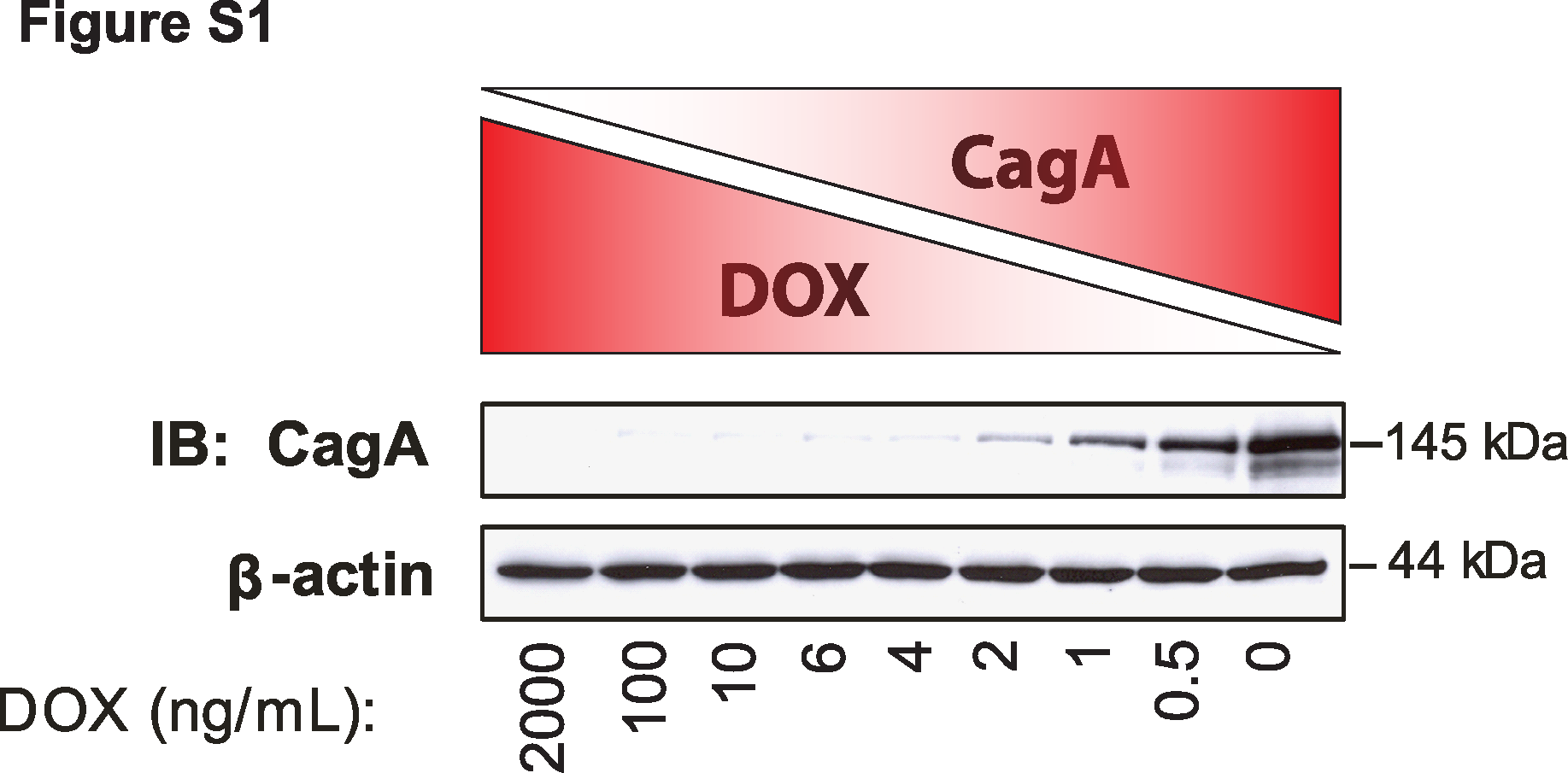

Supplement: Figure S1 — CagA inducible expression in doxycycline repressible Tet-OFF MKN28 cells. The Tet-OFF system was used to inducibly express WT-CagA and phosphorylation resistant (PR) CagA mutant cDNAs in human gastric epithelial MKN28 cells. In the Tet-Off system, transcription of the gene of interest is repressed in the presence of the tetracycline analogue, doxycycline (DOX) and maximally expressed in the absence of DOX. In the panel above, stably transfected, WT-CagA inducible Tet-OFF MKN28 cells were treated with a range of DOX concentrations (0–2000 ng/mL) for 24 hours, total protein lysates obtained and immunoblotted (IB) with a specific antibody to CagA. The immunoblot results show that WT-CagA protein expression is induced with decreasing DOX concentration. Similar results were obtained in PR-CagA inducible Tet-OFF MKN28 cells (data not shown). The β-actin immunoblot verifies equivalent total protein loading in all lanes. All experiments described in this study compare cellular responses between non-induced CagA (2000 ng/mL DOX) and fully induced CagA (no DOX treatment). (TIF) [file pone.0030786.s001.tif]

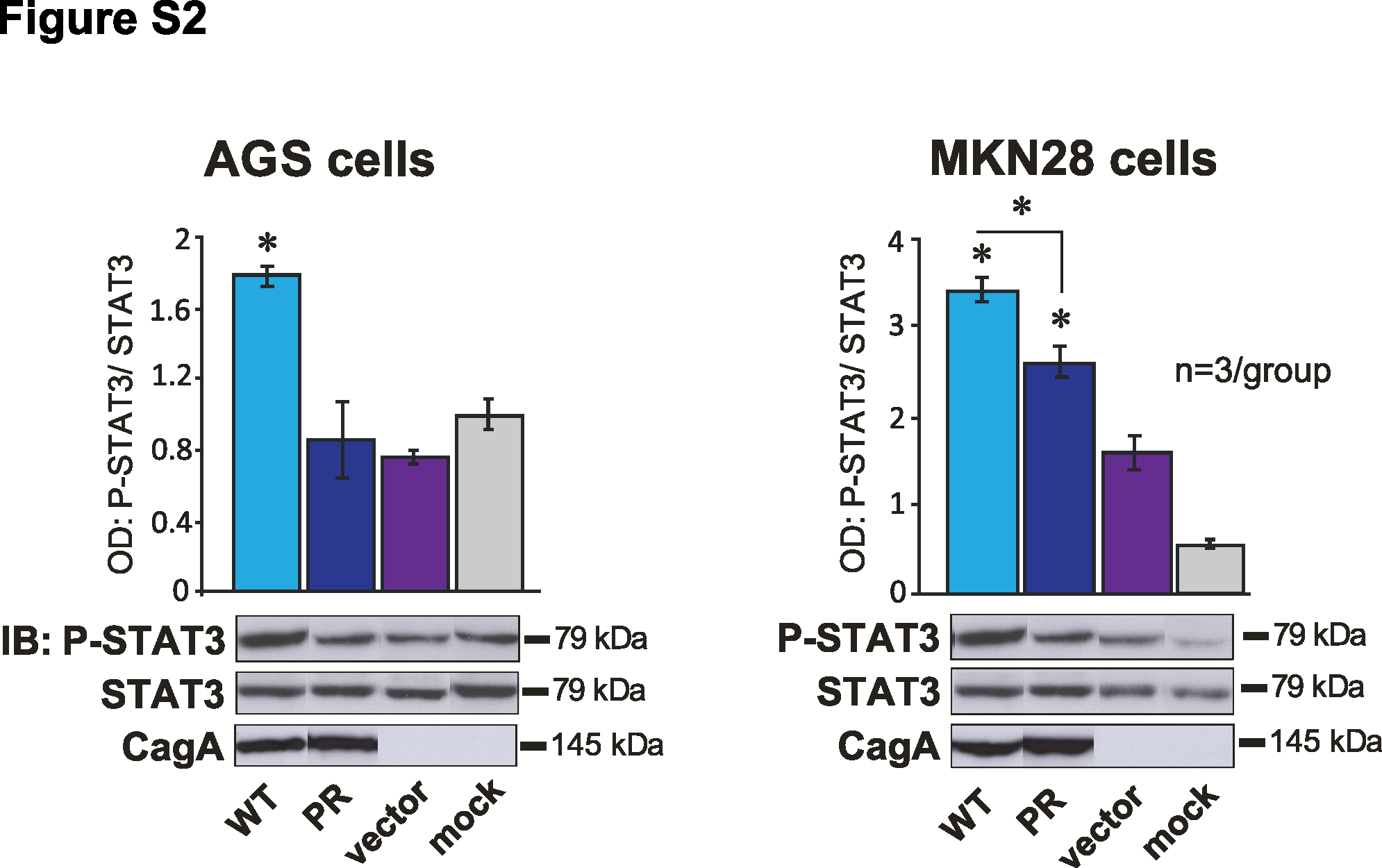

Supplement: Figure S2 — STAT3 activation following transient overexpression of CagA protein. Unmodified AGS and MKN28 cells were transiently transfected with constructs carrying either WT-CagA, or PR-CagA cDNAs driven by the constitutive SR-alpha (SV40/R-U5 T-cell leukaemia virus) promoter fragment, empty vector or mock transfected. Total cellular protein lysates were collected at 48 hours post transfection and immunoblotted for phosphorylated (P)-STAT3, total STAT3 and CagA proteins. Histograms show mean optical densities of P-STAT3 protein bands normalized to total STAT3 protein bands. Protein bands from one randomly selected replicate experiment are shown (from a total of n = 3 replicates/group used to generate the histogram data). Protein molecular weights (kDa) are indicated to the right of the immunoblot images. Error bars (±SEM). Where present, asterisks indicate statistical significance (P<0.05). (TIF) [file pone.0030786.s002.tif]

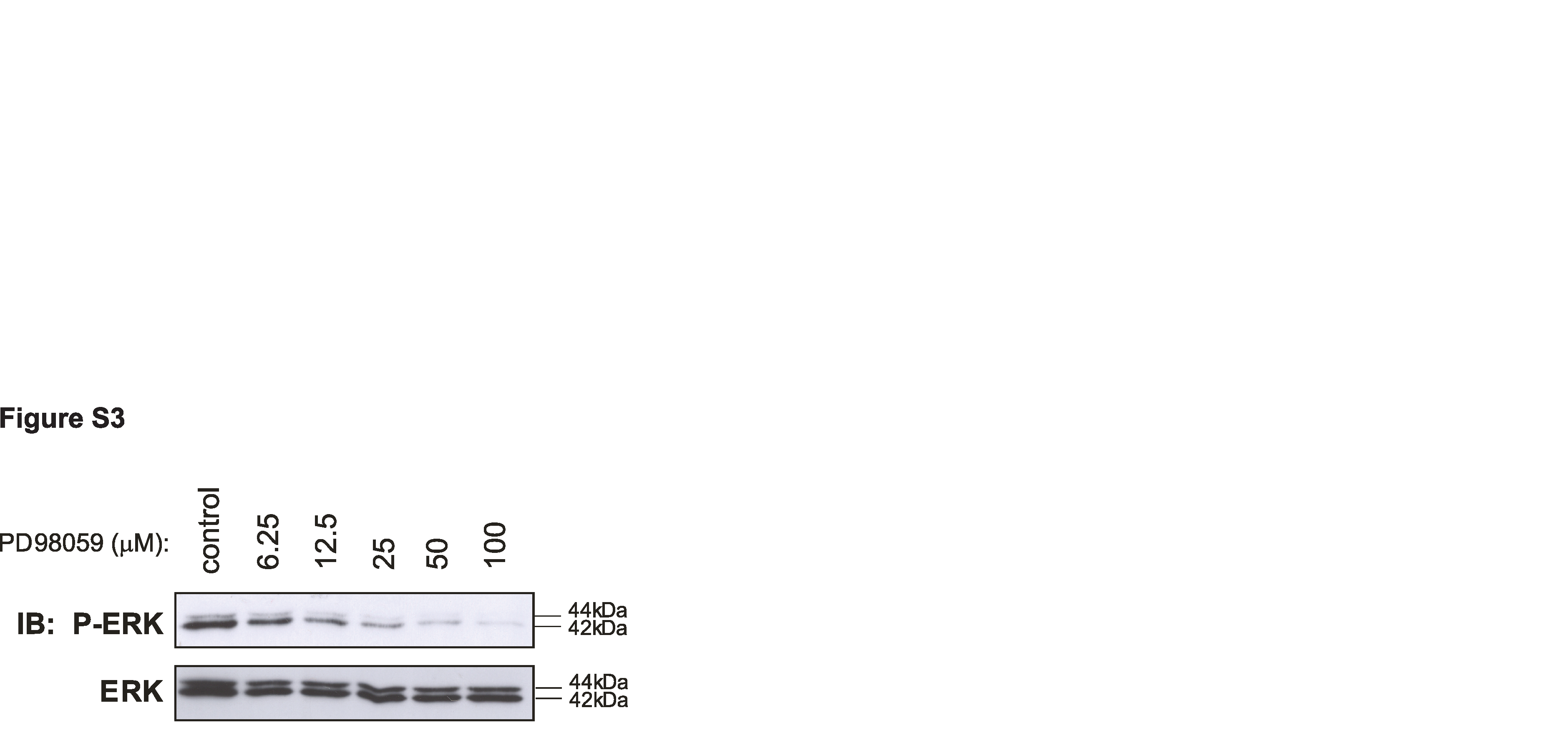

Supplement: Figure S3 — Optimisation of PD98059-mediated ERK inhibition. Tet-OFF MKN28 WT-CagA inducible cells were treated with different concentrations (0–100 µM) of the MEK (proximal activator of ERK) inhibitor PD98059 for 24 hours. Control cells received an appropriate volume of DMSO carrier. To determine ERK activation levels in response to PD98059 treatment, cell protein lysates were obtained and immunoblotted (IB) for total and phosphorylated (P) forms of ERK. Molecular weight of protein bands is indicated (kDa). Treatment with 50 µM PD98059 was the minimum concentration required for sustained ERK inhibition. (TIF) [file pone.0030786.s003.tif]

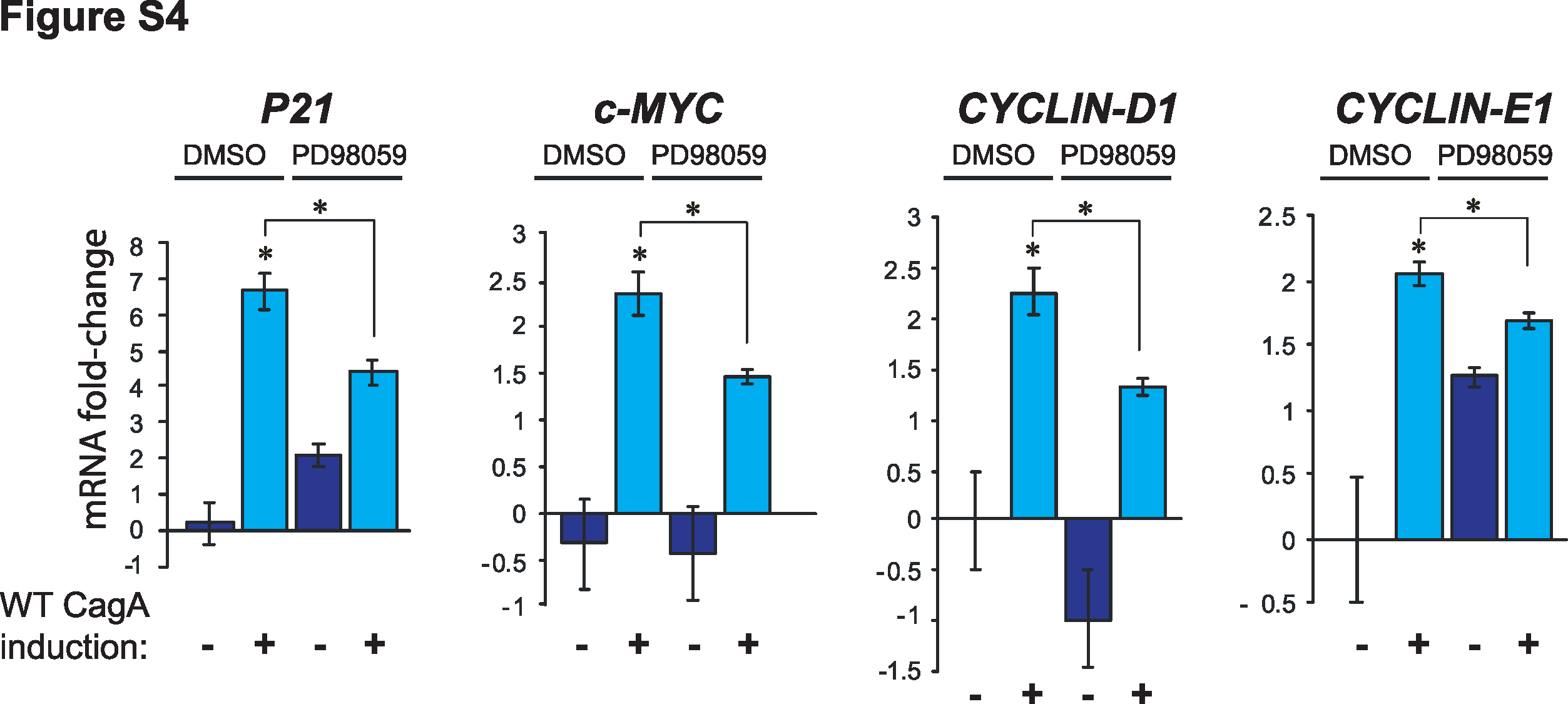

Supplement: Figure S4 — CagA-dependent induction of cell cycle regulator genes following ERK signalling blockade. Quantitative (Q) RT-PCR analysis of P21, cMYC, CYCLIN-D1 and CYCLIN-E1 mRNA expression in CagA expressing (+) and non-induced control cells (−) treated with 50 µM PD98059 or mock treated (DMSO). Histograms show the mean mRNA fold-change compared to non-induced, mock treated cells. Error bars (±SEM). Where present, asterisks indicate statistical significance (P<0.05). (TIF) [file pone.0030786.s004.tif]

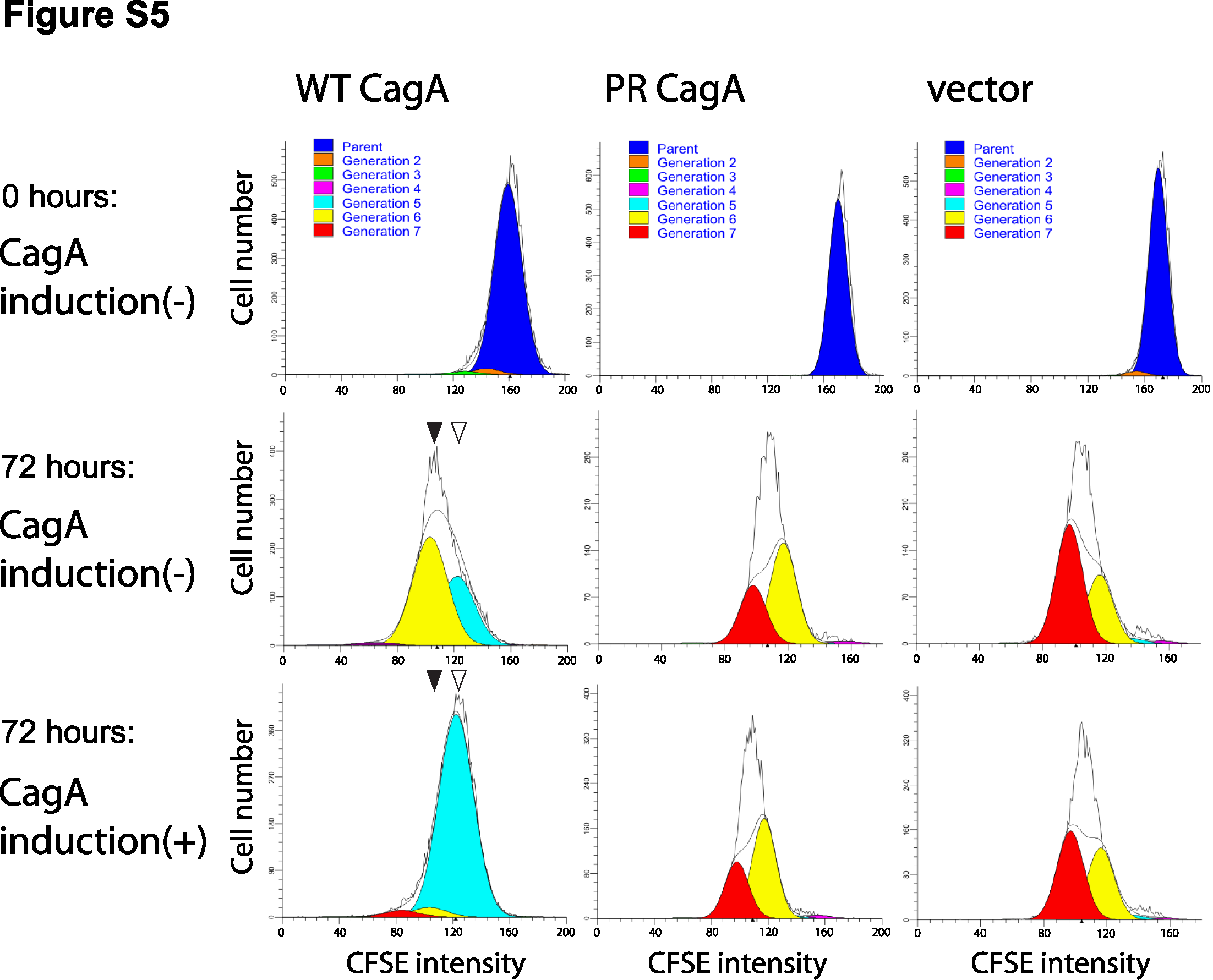

Supplement: Figure S5 — CagA tyrosine phosphorylation leads to growth inhibition of gastric epithelial cells. CagA induction assay showing CFSE proliferation profiles generated by flow cytometric analysis of WT-CagA and PR-CagA expressing cells together with empty vector control cells. The uppermost panels plot initial numbers of CFSE labelled cells at 0 hours (Parent generation), whilst the middle and lower panels plot cell numbers against CFSE intensity signals for non-induced controls (−) and CagA expression induced (+) cells respectively after 72 hours in culture. Successive cell generations from 1(parent) to 7 are indicated by the colour key. White and black arrowheads in the WT-CagA panels respectively illustrate the differential in CFSE fluorescence intensity between induced cells which have arrested in generation 5 and non-induced cells which have continued to divide. (TIF) [file pone.0030786.s005.tif]

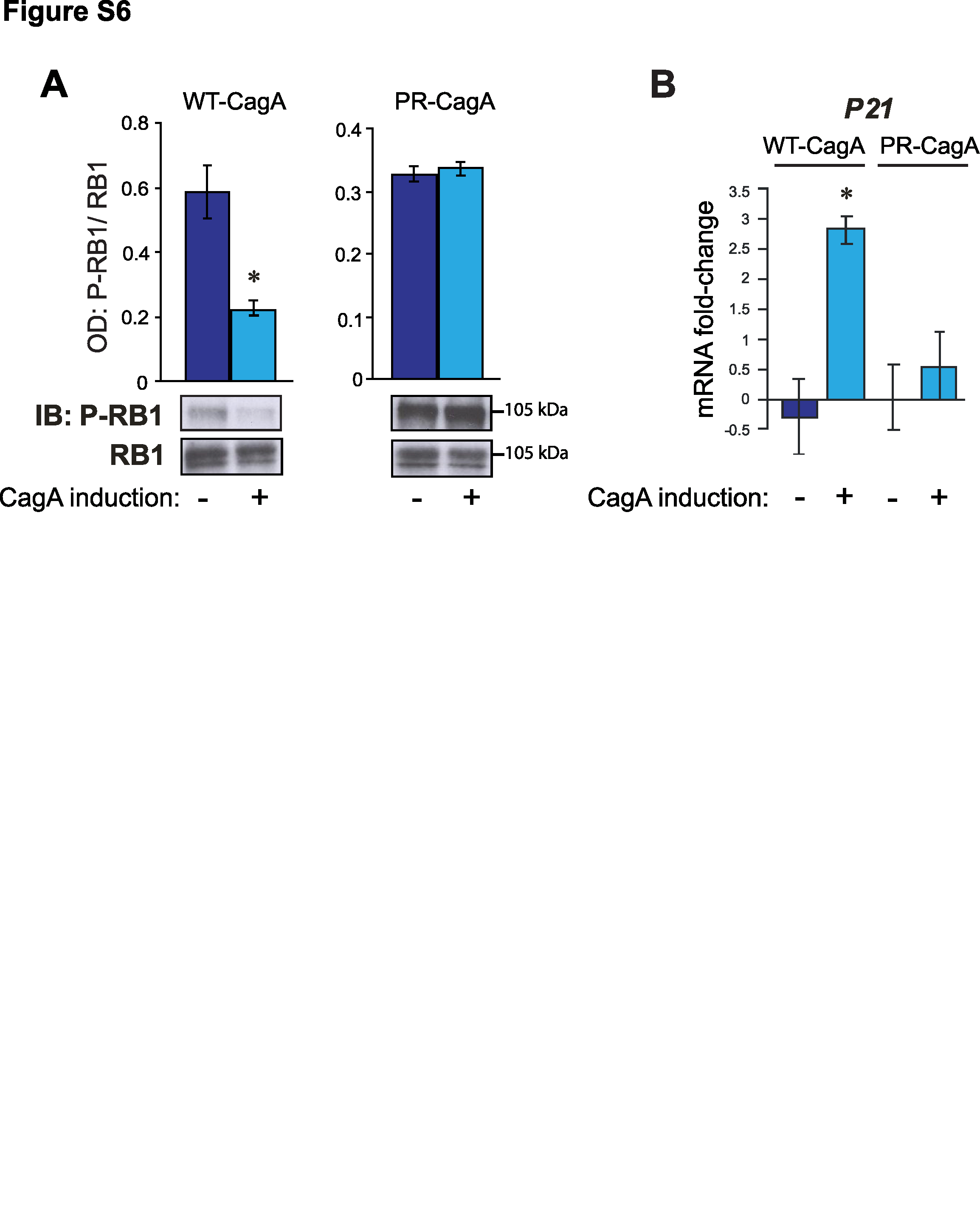

Supplement: Figure S6 — CagA tyrosine phosphorylation deregulates the cell cycle modulators RB1 and P21. (A) CagA induction assay showing immunoblot (IB) analysis of Retinoblastoma protein (RB)1 activity. Histograms show mean fold-change in optical density (OD) of phosphorylated (P)-RB1 protein bands normalized to total RB1 protein in WT-CagA and PR-CagA expressing MKN28 cells compared to non-induced control cells. Blot images from one experiment are shown (from a total of n = 6 replicate cultures/group used to generate the histogram data). (B) QRT-PCR analysis of P21 mRNA expression. Histograms show the mean mRNA fold-change in WT-CagA and PR-CagA expressing cells compared to the respective non-induced controls. Error bars (+/−SEM). Where present, asterisks indicate statistical significance (P<0.05). (TIF) [file pone.0030786.s006.tif]
